# Influence of Players’ Maximum Running Speed on the Team’s Ranking Position at the End of the Spanish *LaLiga*

**DOI:** 10.3390/ijerph17238815

**Published:** 2020-11-27

**Authors:** Juan Del Coso, Diego Brito de Souza, Víctor Moreno-Perez, Javier M. Buldú, Fabio Nevado, Ricardo Resta, Roberto López-Del Campo

**Affiliations:** 1 Centre for Sport Studies, Rey Juan Carlos University, 28943 Fuenlabrada, Spain; 2 Exercise Physiology Laboratory, Camilo José Cela University, 28943 Madrid, Spain; dbrito@ucjc.edu; 3 Sports Research Centre, Miguel Hernandez University of Elche, 03202 Alicante, Spain; vmoreno@umh.es; 4 Complex Systems Group, Rey Juan Carlos University, 28933 Mostoles, Spain; jmbuldu@gmail.com; 5 Department of Competitions and Mediacoach, LaLiga, 28043 Madrid, Spain; fnevado@laliga.es (F.N.); rresta@laliga.es (R.R.); rlopez@laliga.es (R.L.-D.C.)

**Keywords:** soccer, match analysis, team sports performance, exercise training, velocity

## Abstract

The maximum running speed that a football player can attain during match play has become one of the most popular variables to assess a player’s physical talent. However, the influence of a player’s maximum running speed on football performance has not yet been properly investigated. The aim of this study was to determine the influence of a player’s peak/maximum running speed on the team’s ranking position at the end of a national league. A second aim was to investigate differences in maximum running speed among playing positions. To fulfil this aim, the peak/maximum running speeds of 475 male professional football players were recorded for 38 fixtures of the Spanish first-division league (*LaLiga*) from the 2017–2018 season (7838 data points). Players’ peak running speeds in each match were assessed with a validated multicamera tracking system and associated software (Mediacoach^®^). Players’ maximum running speed was established as the fastest running speed they attained during the entire season. Most players (53.5% of the total) had a maximum running speed in the range of 32.0–33.9 km/h, with only three players (0.6%) with a maximum running speed of over 35.0 km/h. Overall, forwards were faster than defenders and both types of players were faster than midfielders (33.03 ± 1.35 > 32.72 ± 1.32 > 32.08 ± 1.63 km/h; *p* < 0.001). There was no association between teams’ maximum running speed and ranking position at the end of the league (*r* = −0.356, *p* = 0.135). The correlations between teams’ maximum speeds and ranking position were low for defenders (*r* = −0.334, *p* = 0.163), midfielders (*r* = 0.125, *p* = 0.610), and forwards (*r* = −0.065, *p* = 0.791). As a result, the variance in the ranking position at the end of the season explained by a team’s maximum speed was of only 7.5%. Finally, as an average for all teams, players’ peak running speeds remained stable at ~30.7 ± 0.6 km/h throughout the whole season. These results suggest that successful and less successful football teams have squads with players able to obtain similar maximum running speeds during match play throughout the season. Hence, players’ maximum running speeds have a poor association with the team’s ranking position at the end of the Spanish professional national league.

## 1. Introduction

Modern professional football (soccer) is a highly demanding team sport characterized by a succession of high intensity actions performed intermittently. These actions, particularly the ones performed in close proximity to the ball, require high values of speed, strength, power, and agility [1]. However, football performance also depends on a variety of individual technical and tactical skills, and on constant interaction with teammates. Therefore, several physical, technical, and tactical capabilities must be well developed to become a successful professional player [2]. In the last few years, research has shifted the focus of the physical determinants in football, conceding more relevance to anaerobic-based actions instead of the traditional view of football as an aerobic-based team sport. In this regard, high intensity running and sprinting account for only ~10% of the total distance covered during a match [3,4], but high-intensity running is a key element to discriminate between elite football players and players of a lower competitive level [5]. Professional football players have become faster over time [2], with a greater capacity to cover a large volume of running at high intensity during matches [6]. On the other hand, players’ aerobic capacity has slightly decreased over the time [7]. Additionally, through the use of principal component analysis (PCA), a technique to discriminate the physical performance variables which are more relevant for football performance, it has been demonstrated that high speed running is within the variables that best describe the profile of the physical demands during an official match [8,9]. Overall, this information suggests that a player’s capacity to cover a high volume of running at high intensity is a crucial determinant of modern football success.

During a competitive match, football players perform a high intensity action every 30 to 90 s, and each high-intensity action lasts, on average, from 2 to 4 s [10]. Hence, from a physical perspective, players perform short-term but continuous high intensity actions interspersed with recovery periods that may vary depending on the evolution of the game. In elite football, players can perform more than 150 intense actions during a match [4], but most of them are not performed at maximum speed. The number of these actions increases with the level of play [2], varies with the playing position on the field [5] with a higher number of sprints and distance at sprint velocity in wide midfielders than in other positions [11], and rises over the course of a season [12]. Interestingly, straight sprinting is the most frequent action prior to scoring a goal [13] and the possession of a high value of maximum running speed is key for overtaking opponents and winning disputed balls. Furthermore, high values of maximum running speed may also reduce the relative neuromuscular load during a match [14] as any action at a given running speed will represent a lower fraction of a player’s maximum speed. Still, it is important to note that the distance covered at high intensity during the competition is not a unique factor associated to football success. In fact, some researchers have suggested that the contribution of the distance covered at high intensity to overall performance is very limited [15], while the distance covered with the possession of the ball is more relevant [16]. Last, the relevance of high intensity running for performance may vary from match to match due to contextual variables such as match location, match outcome, or the level of the opponent [9].

Match-play situations requiring maximum or near-to-maximum running speeds are rarely produced during the game, although they are performed at critical moments. For this reason, the maximum running speed that a football player can attain during match play has become one of the most popular variables to assess a player’s physical performance. Overall, the mean of maximum sprinting velocity of professional football players is normally between 31 and 32 km/h [5], but there are professional players with running speeds ranging from 29 to 33 km/h [17]. However, the majority of high intensity runs in football are shorter than 20 m, which precludes reaching maximum running speeds. Hence, the value of maximum running speed and the distance that a player could cover at high intensity are sometimes unrelated. For example, wide midfielders and external defenders perform more high-intensity running and sprinting [10], but the fastest players are usually the forwards [18]. To date, the influence of players’ maximum running speed on the overall team’s football performance has not yet been properly investigated. As mentioned above, it is clear that the possession of a high running speed and the capacity to repeat sprints over the time during a competitive match are both potentially beneficial factors for football performance. Nevertheless, to date, it is unknown if it is better to direct players’ physical conditioning to obtain formidable high maximum speeds or to direct training to obtain players able to produce sprints of lower/submaximal velocity but with a higher capacity of repeating them over time. For this reason, the aim of this study was to determine the influence of players’ maximum running speed on the team’s ranking at the end of a national league. This objective was intended to evaluate the relevance of players’ maximum speed on football performance during a national football league. A second aim was to investigate differences in maximum running speed among playing positions. We hypothesized that more successful football teams (i.e., the ones in the first ranking positions at the end of the season) would have squads with the fastest players in all field positions, in comparison to worse-ranked teams. Additionally, forwards will be faster than any other field position.

## 2. Materials and Methods

### 2.1. Participants

The study sample was composed of 475 football players competing in the Spanish first-division football league (*LaLiga*) during the 2017–2018 season. This corresponds to the entire population of professional football players that competed at least for 30 min in the 2017–2018 season. From the total, 175 players were defenders, 196 were midfielders, and 105 were forwards (36.8/41.3/21.9%, respectively). The number of players per team and per playing position in the field, in addition to their maximum running speeds during the season, are detailed in Table 1. Of note, data from the players competing in the team classified in 13th position were not used in this investigation, as none of the matches played in its stadium reported data on running actions during the 2017–2018 season. In accordance with *LaLiga*’s ethical guidelines, this investigation does not include information that identifies football players. The Institutional Review Board of the Camilo José Cela University approved this study, which is in accordance with the latest version of the Declaration of Helsinki.

### 2.2. Procedures

This investigation is a descriptive and comparative analysis to determine the importance of players’ maximum/peak running speeds on football performance. Data were obtained from *LaLiga*, which authorized the use of the variables included in this investigation. The Spanish national first-division football league is composed of 20 teams competing in a total of 38 fixtures (for a total of 380 matches for season). Data from the matches of the team classified in 13th position were excluded from the investigation because the multicamera tracking system was not installed in its stadium during the season under investigation. Hence, this investigation contains data on 361 matches played across 38 fixtures. In each fixture, players’ peak running speed, defined as the highest running speed attained in a particular match, was obtained and recorded for all the field players, for a total of 7838 values across the season. Only peak running speeds of players competing during at least 30 min in the match were considered for analysis to ensure that the players had time to produce a football action at high/peak intensity. Maximum running speed was defined as the highest running speed obtained by a player during the entire season, using all the values recorded by this player during all the matches he participated in for at least 30 min. The data on goalkeepers were excluded due to the different nature of their movement patterns during the game. To determine the influence of players’ peak/maximum running speeds on football performance, a comparison was made of the individual and team average running speeds (1) according to the ranking position at the end of the season and (2) according to ranking categories as follows: the league champion (1st); teams classified for the Champions League (2nd–4th); teams classified for the Europa League (5th and 6th); teams in the middle of the ranking (7th–17th); and the relegated teams (18th–20th). An analysis of teams’ maximum speeds depending on the playing position was also performed by using three positions: defenders, midfielders, and forwards.

### 2.3. Instrument

Data on peak/maximum running speeds were extracted using the match statistics software Mediacoach^®^ (*LaLiga*, Madrid, Spain), a multicamera tracking system that can accurately assess the instantaneous running speed of all the players on the field. Briefly, Mediacoach^®^ records the position of each player at 25 frames per second using a stereo multicamera system composed of two multicamera units placed at either side of the midfield line. Each multicamera unit contains three cameras with a resolution of 1920 × 1080 pixels, which are synchronized to provide a stitched panoramic picture. The panoramic picture is then employed to create the stereoscopic view that allows triangulating of all the players on the field and the ball. In the case of a lack of location of a player due to occlusions by another player, an experienced operator manually corrected the position during measurement. This correction is common in corners and fouls but rarely occurs during actions where players obtained their peak/maximum running speeds. Hence, the manual corrections had minimal relevance for the objectives of this investigation. The validity of this software to assess movement demands during match play has been obtained through high agreement with the data obtained with GPS [19,20] and with data obtained from a reference camera system (i.e., VICON motion capture system [21]).

### 2.4. Statistical Analysis

We set the significance level for the statistical analysis at *p* < 0.05 and all analyses and calculations were performed using the SPSS v.20 software package (IBM, Armonk, NY, USA). Initially, we used the Levene test to verify sample homogeneity and the Kolmogorov–Smirnov test to verify the normality of peak/maximum running speeds. Descriptive means and standard deviations were calculated in each team and for each playing position (Table 1). We used a one-way analysis of variance (ANOVA) of repeated measures to compare peak running speeds among the 38 fixtures that comprised the championship. We used a two-way ANOVA (fixture x ranking category) to determine differences in the evolution of maximum running speed across the season among the ranking groups. The number and distribution of players according to their maximum running speeds were calculated using 1.0 km/h intervals. A two-way ANOVA (playing position x ranking) was used to search for differences among teams in the maximum running speed for any playing position. In the case of a significant F value in the ANOVAs, the differences between groups were identified by Tukey post hoc tests. For the differences in maximum running speed between playing positions, the effect size was calculated in Cohen’s *d* units [22]. Pearson’s correlation coefficients (*r*) were used to assess the association between a team’s maximum running speed and ranking position at the end of the season. The size of a correlation coefficient was evaluated following Hinkle et al. [23]. Then, a multiple regression analysis was carried out in a stepwise interactive mode to assess the influence that a team’s maximum running speed had on the ranking position the end of the league. In the regression analysis, all match statistics were introduced based on their correlation with the residual (*p* < 0.1) and their intercorrelation with variables that already existed in the equation. The r^2^ values were adjusted for the number of cases and parameters included in the analysis [24].

## 3. Results

**Figure 1:**
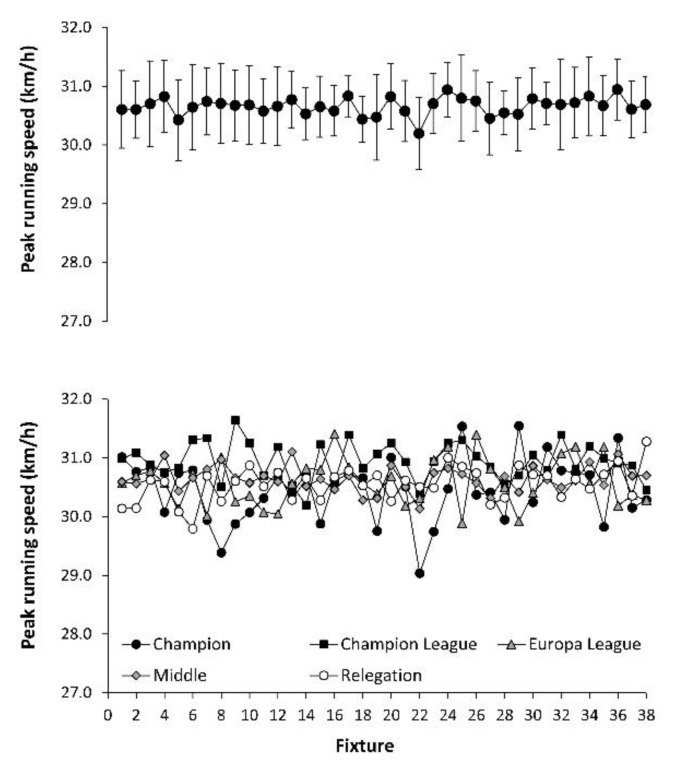
Peak running speed (**upper panel**) and peak running speed in teams with different ranking categories (**lower panel**) across *LaLiga* 2017–2018.

Figure 1 depicts the peak running speeds obtained by the football teams during the season. In the upper panel, the data include the mean of all teams competing in *LaLiga* in each of the 38 fixtures that comprised the championship, and the one-way ANOVA revealed no statistically significant differences in the values of peak running speed among the different fixtures (F = 1.282; *p* = 0.372). In the lower panel, peak running speeds are presented according to different ranking groups. The two-way ANOVA revealed no main effect of the ranking group in the maximum running speeds obtained during the season (F = 2.191; *p* = 0.134).

**Figure 2:**
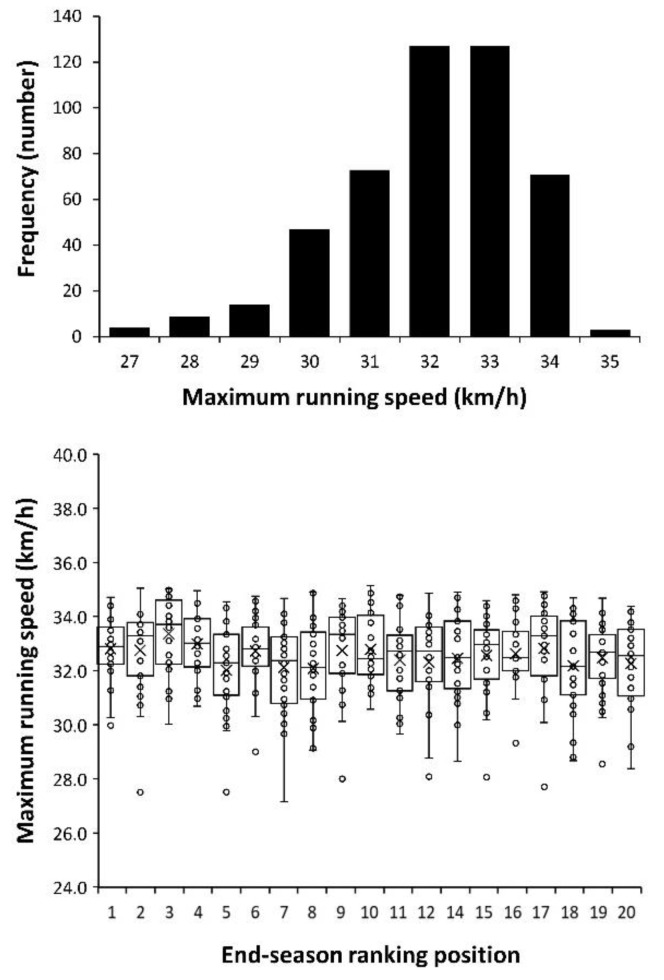
Number of players according to their maximum running speed (**upper panel**) and individual maximum running speed according to the end-season ranking position of the teams competing in *LaLiga* 2017–2018 (**lower panel**). Data represent the maximum running speed obtained by each player in the 2017–2018 season. Note: There were no data for the team classified in 13th position.

The number of players distributed according to their maximum running speeds during the 2017–2018 season is presented in Figure 2. Most players (53.5%) were in the range of 32.0–33.9 km/h, with 71 players (14.9%) surpassing 34.0 km/h and only 3 players (0.6%) with maximum running speeds of over 35.0 km/h. Still, there were 27 players (5.7%) who did not reach 30.0 km/h during the competitive season. Nevertheless, teams’ maximum speeds were unrelated to the end of season ranking position obtained, as the one-way ANOVA revealed no differences in the maximum running speed values among the different teams competing in *LaLiga* (F = 1.308; *p* = 0.177). Additionally, the correlation coefficient between teams’ maximum speeds and ranking position was low (r = −0.356, *p* = 0.135).

**Figure 3:**
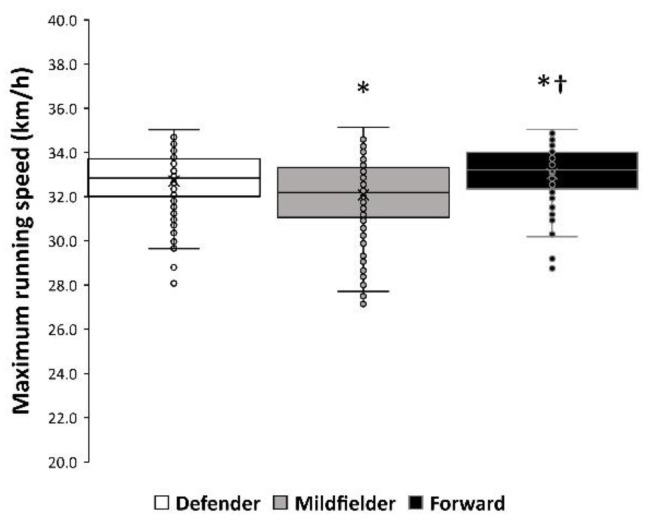
Individual maximum running speed according to the playing position in the field in *LaLiga* 2017–2018. (*) Different from defender at *p* < 0.05. (†) Different from midfielder at *p* < 0.05.

There was a main effect of the playing position on maximum running speed (F = 18.765; *p* < 0.001). Overall, forwards were the fastest players (33.03 ± 1.35 km/h) with a higher maximum running speed than defenders (32.72 ± 1.32 km/h; *p* = 0.025, *d* = 0.23) and midfielders (32.08 ± 1.63 km/h; *p* < 0.001, *d* = 0.63). Defenders were also faster than midfielders (Figure 3; *p* < 0.001, *d* = 0.43). However, there was not any interaction between the ranking position of the team and the playing position (Table 1; F = 0.897; *p* = 0.643). The correlation coefficient between teams’ maximum speeds and ranking position was low for defenders (r = −0.334, *p* = 0.163) and small for midfielders (r = −0.125, *p* = 0.610) and forwards (r = −0.065, *p* = 0.791). Finally, the variance in the ranking position obtained at the end of the league as explained by the team’s maximum speed was of only 7.5% (contribution *r*^2^ adjusted = 0.075, *p* = 0.427).

## 4. Discussion

With the incorporation of microtechnology into elite football (mainly, the use of Global Positioning System devices and multicamera tracking systems), sport scientists and physical trainers are now analyzing a high number of physical and physiological variables that may have the potential to contribute to overall football performance. This represents, in most cases, an excess of data that complicates the understanding of what variables are important for the game [8]. Additionally, the existence of a high number of variables may lead to oversimplification of the game by using them to categorize players. In this regard, the maximum running speed that a player can obtain during a match has become a widely used variable to assess a player’s physical talent, despite the evidence to argue that this variable is important for a player’s and team’s performance being scarce. Peak/maximum running speed represents one single action during the match, while professional football players perform more than 150 intense actions during match play [4]. Hence, the potential evaluation of a player’s physical talent, by using only one action during match play, may lead to incorrect assumptions, at least in elite football. Despite the popularity of this performance variable, we are not aware of any previous investigations that have aimed to determine the influence of players’ peak/maximum running speeds on the team’s overall football performance. The current investigation presents an analysis of the fluctuations of peak running speeds obtained during matches throughout a complete season of *LaLiga*. In addition, the players’ maximum running speeds have been compared to the ranking obtained at the end of the championship, while differences in maximum running speed among playing positions have been analyzed. Overall, the current investigation demonstrates that peak running speed was maintained relatively constant throughout the championship, a characteristic shared by the Champion, the teams classified for the Champions League, the teams classified for the Europa League, middle teams, and the relegated teams (Figure 1). Additionally, all teams competing in *LaLiga* had squads with comparable maximum running speeds, irrespective of their ranking position at the end of the championship (Figure 2). The similarity in maximum running speeds among teams was equally present in defenders, midfielders, and forwards (Table 1), although forwards were the fastest players in each team (Figure 3). In addition, the correlation coefficient between teams’ maximum speed and ranking position was low and the variance in the ranking position obtained at the end of the league explained by team’s maximum speed was of only 7.5%. Together, this information points towards a poor association between players’ maximum/peak running speeds and the team’s overall football performance during a national league. This notion does not dispute the importance of covering high volumes at high intensity for football performance but suggests that most, if not all, professional teams in *LaLiga* possess players able to reach over 30 km/h, limiting the discriminatory utility of maximum running speeds to distinguish between better- and worse-ranked teams.

Recently, it has been found that football teams competing in a national football league needed 8–10 fixtures from the beginning of the season until they reached a plateau in match running performance [12]. The necessity of competing in 8–10 matches before reaching a steady-state physical performance was evident for the running distance at over 24 km/h and for the number of running actions performed above this threshold. However, the current analysis indicates that on average for all the teams competing in *LaLiga*, players’ peak running speeds were 30.6 ± 0.7 km/h for the first fixture, and a comparable value was obtained throughout the competition (Figure 1). This result suggests that professional football players are able to reach maximum or near-to-maximum running velocities from the first competitive match, even when they are not ready to perform a large volume of high intensity running. While maximum running speed during a match is mainly related to mechanical determinants aimed to produce great vertical ground reaction forces per unit of body mass [25,26], the capacity to produce a high amount of running actions at high intensity is more related to metabolic parameters such as the capacity to supply energy from different pathways during the running action and during the recovery, and the ability to reduce the intramuscular accumulation of metabolic by-products [27,28]. Therefore, it seems that professional football players possess the mechanical capacity to perform at least one running action at very high speed from the beginning of the championship, but they need several fixtures to obtain the physiological adaptations to produce high values of running distance at high intensity and sprinting velocities.

Peak running speed and the amount of running performed at high intensity are physical variables that represent different performance outcomes during a match [17]. Peak running speed is normally obtained during an offensive or defensive football action without the ball and in a field position that allows the distance necessary to obtain appropriate acceleration and maximum velocity. Players obtain their peak running speed during a critical action of the game but this represents only one of the hundreds of high intensity actions and dozens of sprints performed during a match [2,4]. Accordingly, while several previous investigations have coincided in establishing the importance of high intensity running during a match for overall football performance [2,4,5], the current investigation suggests that players’ peak/maximum running speeds are comparable in all teams competing in *LaLiga*, irrespective of their ranking and competitive level.

In this regard, the distance covered at over 21 km/h during a national league has a modest capacity to discriminate between successful and less successful teams, especially if the distance is covered with the ball [16]. However, the utility of the physical demands during a match to predict football performance is lower when compared to match statistics such as shooting accuracy, the number of shots performed, and the capacity to prevent the rival from shooting [29,30]. All this information points towards a poor capacity of teams’ maximum running speeds to anticipate the football performance of a squad. From a practical perspective, this information also suggests the convenience of focusing training on more useful determinants of football performance like the development of a high capacity to repeat sprints during a match and tactical and strategic interventions to enhance shooting efficacy, reducing the time devoted to improving players’ maximum running speeds.

The upper panel in Figure 2 indicates that 94.3% of players (448 out of 475) competing in *LaLiga* are capable of running at over 30.0 km/h. Only three players were able to run at over 35.0 km/h, while the fastest players reached 35.2 km/h. Overall, most players were able to obtain sprinting velocities between 32 and 33 km/h (Figure 2). Although these values of maximum running speed are excellent for football players [5,17], they are much lower than the peak velocity obtained by elite-level athletes during sprint events [2]. Furthermore, the presence of this high number of players running at over 30 km/h hinders the capacity of using maximum speed actions to overcome rivals during match play. Midfielders and defenders perform more high-intensity running and sprinting [10] but, as previously suggested, the fastest players are usually the forwards [18]. This pattern was found in most teams in the present investigation (Table 1) and suggests that the playing field position of a defender has evolved to become a position with fast players to increase their aptitude to defend against fast forward players, and vice versa.

The current investigation presents some limitations that should be discussed to improve the applicability of its outcomes to overall football performance. First, this investigation contains a notational analysis of peak/maximum running speed in a sample of professional football players competing in a national league (*LaLiga*). By using different statistics (e.g., simple and multiple correlations and groups comparison), we have contrasted team peak/maximum running speed of successful and less successful football teams in *LaLiga*. While this analysis is useful to understand the relevance of players’ maximal running speed on the ranking position obtained in the competition, football performance is a complex construct that is influenced by a myriad of intrinsic and extrinsic factors, the one analyzed here being only one of them. In fact, it may be argued that the current analysis is reductionist because it omits the “why”, “where”, and “how” of the actions that lead to peak/maximum running speed [31]. In this regard, football actions requiring maximum or peak running speeds represent a low portion of the total number of high-intensity actions executed during a match, but the context in which they are performed is critical because it demands the player to obtain his/her maximal effort. Future investigations with more ecological approaches should be carried out to establish the relevance of peak/maximum running speeds in a more complex dynamic environment [32], including the cause of the sprint action, the location on the pitch of the sprint action, the main outcome of the sprint action, and the interactions of the player performing the sprint action with his/her teammates and rivals.

From a practical perspective, and of the opinion of the authors of the current research, physical training in elite football should be more focused on enhancing the ability to repeat sprints of sub-maximum intensity (e.g., between 21 and 30 km/h) to obtain high volumes of running distance at >24 km/h, rather than on improving players’ maximum running speed. This is important as the training routines used for such objectives may be substantially different. Additionally, a key portion of physical and conditioning training should be devoted to increasing a player’s capacity to accelerate/decelerate in short distances as they perform four times as many accelerations as reported sprints per match [33]. Lastly, the physical training devoted to developing maximum running speed could be focused on ensuring that players obtain at least 30–32 km/h of peak velocity during match play, as this is the peak running speed that most players produce during a game. The obtaining of higher velocities will likely impact on a few actions during the game, but as suggested by the results of this study, they will have low influence on the overall team performance at the end of the league. Of note, the utility of possessing a team squad with players able to obtain high peak/maximum running speeds may depend on the playing style. It is probable that teams with direct play when attacking and exerting high pressure while defending may benefit from faster players.

## 5. Conclusions

In summary, successful and less successful football teams competing in *LaLiga* have squads with players able to obtain similarly high maximum running speeds during match play. In addition, players of successful and less successful teams are capable of obtaining peak running speeds from the first fixture of the competition and maintain it across the season. Although football is a sport with a relatively low number of goals, and the goals are habitually preceded by power and speed actions [13], players’ maximum running speeds had minimal impact on the team’s ranking position at the end of the Spanish national league. In fact, the variance in the ranking position obtained at the end of the season explained by the team’s maximum speed was of only 7.5%.

## Figures and Tables

**Table 1:** Number of players and players’ maximum running speed according to the team’s ranking in the 2017–2018 season of *LaLiga* championship.

Ranking	Total	Defender	Midfielder	Forward
1st	22 32.8 ± 1.2	9 33.1 ± 1.0	8 32.0 ± 1.3	5 33.5 ± 1.0
2nd	23 32.8 ± 1.6	8 33.1 ± 1.1	9 31.8 ± 2.0	6 33.8 ± 0.7
3rd	21 33.4 ± 1.5	8 34.1 ± 1.0	9 32.8 ± 1.5	4 33.1 ± 1.8
4th	23 33.0 ± 1.2	9 33.0 ± 0.5	11 32.9 ± 1.5	3 33.9 ± 1.4
5th	27 32.0 ± 1.6	9 32.2 ± 1.5	12 31.5 ± 1.7	6 32.9 ± 1.3
6th	24 32.7 ± 1.3	9 33.2 ± 0.8	7 32.5 ± 0.8	8 32.4 ± 1.9
7th	28 32.1 ± 1.7	10 32.0 ± 1.3	13 31.9 ± 2.0	5 32.8 ± 1.4
8th	26 32.1 ± 1.6	10 32.1 ± 1.5	9 31.0 ± 1.4	7 33.5 ± 1.2
9th	23 32.8 ± 1.6	8 32.8 ± 1.4	11 32.6 ± 2.0	4 33.1 ± 0.9
10th	20 32.8 ± 1.3	8 33.0 ± 1.5	8 32.3 ± 1.4	4 33.3 ± 0.8
11th	23 32.4 ± 1.4	8 32.4 ± 1.7	11 32.0 ± 1.2	4 33.4 ± 1.0
12th	22 32.3 ± 1.7	9 32.2 ± 2.1	7 32.4 ± 1.2	6 32.4 ± 1.9
14th	27 32.5 ± 1.5	8 32.8 ± 1.4	12 32.1 ± 1.7	7 32.6 ± 1.3
15th	29 32.6 ± 1.4	9 33.3 ± 0.7	11 32.5 ± 1.8	9 31.9 ± 1.1
16th	23 32.6 ± 1.2	11 32.9 ± 1.1	8 31.9 ± 1.1	4 33.3 ± 1.1
17th	23 32.8 ± 1.7	9 32.9 ± 1.1	10 32.2 ± 2.1	4 34.3 ± 0.7
18th	27 32.2 ± 1.8	11 32.1 ± 1.7	13 31.8 ± 1.8	3 34.1 ± 0.2
19th	33 32.5 ± 1.3	11 32.6 ± 1.0	15 31.9 ± 1.4	7 33.4 ± 0.8
20th	31 32.3 ± 1.5	11 32.3 ± 1.2	12 32.0 ± 1.8	8 32.6 ± 1.7

Note: There were no data for the team classified in 13th position.
